# Down-regulation of microRNA-23a promotes pancreatic ductal adenocarcinoma initiation and progression by up-regulation of FOXM1 expression

**DOI:** 10.1016/j.gendis.2023.101203

**Published:** 2023-12-22

**Authors:** Lixin Liang, Tian Cai, Xiaojia Li, Jianhong An, Sen Yu, Yang Zhang, Fengjie Guo, Fang Wei, Jie He, Keping Xie, Tingting Jiang

**Affiliations:** a Center for Pancreatic Cancer Research, South China University of Technology School of Medicine, Guangzhou, Guangdong 510006, China; b Department of Laboratory Medicine, The Sixth Affiliated Hospital and Nanhai People's Hospital, South China University of Technology School of Medicine, Foshan, Guangdong 528200, China; c The Second Affiliated Hospital and Guangzhou First People's Hospital, South China University of Technology School of Medicine, Guangzhou, Guangdong 510006, China

**Keywords:** FOXM1, Gene expression, Gene regulation, MicroRNA, Pancreatic cancer, Targeted therapy

## Abstract

Transcriptional factor Forkhead box M1 (FOXM1) plays an important role in pancreatic ductal adenocarcinoma (PDAC) development and progression. The molecular mechanisms underlying its dysregulation remain unclear. We identified and functionally validated the microRNAs (miRNAs) that critically regulate FOXM1 expression in PDAC. The expression levels of miRNA-23a (miR-23a-3p and -5p) were altered in PDAC cell lines and their effects on FOXM1 signaling and cell proliferation and migration and tumorigenesis were examined *in vitro* and *in vivo* using mouse PDAC models. Compared with non-tumor pancreatic tissues, PDAC tissues and cell lines exhibited significantly reduced levels of miR-23a expression. Reduced miR-23a expression and concomitant increase in FOXM1 expression were also observed in acinar-to-ductal metaplasia and pancreatic intraepithelial neoplasia, the major premalignant lesions of PDAC. Transgenic expression of miR-23a reduced the expression of FOXM1 and suppressed cell proliferation and migration in PDAC cells, whereas the inhibitors of miR-23a did the opposite. Loss or reduced levels of miR-23a increased the levels of FOXM1 expression, while increased expression of FOXM1 down-regulated miR-23a expression, suggesting that miR-23a and FOXM1 were mutual negative regulators of their expression in PDAC cells. Therefore, the miR-23a/FOXM1 signaling axis is important in PDAC initiation and progression and could serve as an interventional or therapeutic target for patients with early or late stages of PDAC.

## Introduction

Pancreatic ductal adenocarcinoma (PDAC) is a lethal malignancy. Although surgical removal is the ideal treatment for PDAC, most patients are poor candidates for resection due to late diagnosis.^1^ Gaining a deeper comprehension of the molecular foundation of PDAC pathogenesis is crucial for designing inventive approaches to prevention, early detection, and treatment. It is well-known that PDAC has a wide range of alterations in molecular signaling cascades, including mutations in KRAS, TP53, CDKN2A, and SAMD4, which are major factors for the initiation and progression of PDAC.^2^^,^^3^ Other molecules also have been identified as potential diagnostic and therapeutic biomarkers in PDAC, such as Kruppel-like factor 4 and Forkhead box M1 (FOXM1).^4^^,^^5^ FOXM1, as a member of the Forkhead box transcription factor superfamily, is critically important to the proliferative ability of cells and the maintenance of self-renewal for cancer stem cells.^6^, ^7^, ^8^

One important mechanism that FOXM1 acts as a critical biomarker in the development and progression of PDAC, is the exuberant signaling through its overexpression. FOXM1 regulates the expression of numerous genes crucial to various physiological processes and diverse facets of tumor biology.^5^^,^^9^ Recent research has established a connection between elevated FOXM1 expression and unfavorable prognoses in various cancer types, including PDAC, identifying it as an oncogenic transcription factor.^5^^,^^10^, ^11^, ^12^, ^13^ FOXM1 exerts regulatory control over various dimensions of PDAC, encompassing the cell cycle process, cellular proliferation, cell differentiation, repair of DNA damage, programmed cell death, angiogenesis, metastatic spread, tissue homeostasis, stem cell functions, effects on therapeutic drugs, and unfavorable prognostic indicators.^4^^,^^5^ FOXM1 executes its functions through multiple mechanisms, including inhibiting mitosis through suppression of the spindle assembly checkpoint and down-regulation of Cyclin B expression^14^; binding to the PD-L1 promoter, leading to the selective upregulation of PD-L1 expression^15^; binding to β-catenin and mediating its nuclear translocation,^16^ involved in the aberrant activation of PI3K signaling pathway and inactivation of TP53,^17,18^ transactivation of the uPAR-caveolin-1 signaling pathway, and promotion of epithelial-to-mesenchymal transition^9^^,^^19^; and forming a positive feedback loop with HGF/Met,^20^ attenuating the tumor suppressive role of merlin and enhancing Wnt/β-catenin signaling.^21^ Furthermore, FOXM1 engages in interactions with the signaling cascades regulating pancreatic cancer stem cells, eliciting the up-regulation of cell surface biomarkers such as CD44 and EpCAM.^22^ Therefore, FOXM1 exerts a substantial role in the initiation, progression, and metastatic processes of PDAC.

The molecular mechanisms accounting for the elevated expression of FOXM1 have not been fully elucidated. KRAS mutation facilitates the translocation of FOXM1 into the cell nucleus, leading to the augmentation of target gene expression.^23^ The overactivation of PI3K and the loss of TP53 function are causally associated with the up-regulation of FOXM1.^17^^,^^18^ Epigenetic changes also contribute to the disruption of the FOXM1 signaling pathway in PDAC.^24^ Serving as a pivotal contributor to epigenetic modifications, microRNAs (miRNAs) have significant effects on the activation of FOXM1 signaling.^25^ Down-regulation of certain miRNA expression is linked to the overexpression of FOXM1. FOXM1 and miRNAs also elicit divergent biological effects on different cell types.^25^^,^^26^ It is imperative to ascertain the specific miRNAs that regulate FOXM1 signaling expression and profoundly influence PDAC pathogenesis.

In this study, our objective was to explore the upstream mechanisms responsible for the dysregulated FOXM1 function in PDAC. Our results revealed a novel inhibitory function of miR-23a in modulating the activation of FOXM1 in PDAC cells, exerting a functionally suppressive influence on these cells. Furthermore, we observed that elevated FOXM1 expression contributes to the down-regulation of miR-23a, suggesting a reciprocal negative regulatory relationship between FOXM1 and miR-23a.

## Materials and methods

### Patient samples and immunohistochemistry

With approval from the Ethics Committee, tissue microarrays containing samples from the PDAC patients undergoing surgical resection with histologic diagnosis of PDAC (Shanghai Company, China) were used. All investigations involving human participants adhered to the principles outlined in the Declaration of Helsinki. Whenever required, written informed consent was acquired from all individuals involved. Immunohistochemistry for FOXM1 of PDAC patient samples was performed using a primary FOXM1 antibody, and a DAB substrate kit [GK500710, Gene Tech (Shanghai) Company Limited, China] with species-specific anti-rabbit and anti-mouse IgG. Pancreatic amylase, CK19, α-SMA, and desmin were also analyzed, and detailed information on antibodies can be found in Table S1. The immunohistochemical reactivity of FOXM1 was scored by two working pathologists according to the modified method as described previously.^27^ FOXM1 immunoreactivity was categorized as weak (1), moderate (2), or strong (3). Second, the different percentages of FOXM1-reactive tumor cells were classified based on the following cutoff levels acquired by light microscope and ImageJ software: “1” score was given for faint or partial staining intensity with less than 10% of the tumor cells stained, or no visible staining, defined as “negative/weak”; “2” score was given for moderate staining intensity with >10%–50% of the tumor cells stained positive, defined as “moderate”; and “3” score for strong staining intensity with >50%–100% of tumor cells stained positive, defined as “strong”.^9^

### Quantitation of miRNAs isolated from microdissected cells identified through immunostaining

Formalin-fixed, paraffin-embedded PDAC specimens obtained from treatment-naive patients who underwent surgery were subjected to processing for FOXM1 staining. The immunohistochemical staining for FOXM1 was performed using a previously described anti-FOXM1 antibody (Table S1).^16^ To ensure specificity and prevent stromal cell contamination, laser-assisted microdissection (CellCut Plus, Molecular Machines & Industries, Eching, Germany) was utilized to isolate tumor cells positively expressing FOXM1.

Small RNA-enriched total RNA was extracted from cells with a Recover All Total Nucleic Acid Isolation Kit, following the guidelines provided by the manufacturer. Briefly, 3.5-mm sections of formalin-fixed, paraffin-embedded PDAC specimens were treated with xylene to remove paraffin. Subsequently, the tissue underwent protease and DNase treatment at 50 °C. Following thorough rinsing, the total RNA, encompassing all the miRNA fraction, was extracted. The concentrations and integrity of the isolated RNA were evaluated utilizing a NanoDrop One/OneC spectrophotometer (Thermo Fisher Scientific, Waltham, MA).

### Mice

*KC* mouse models were generated in-house using *LSL-Kras*^*G12D/+*^ and *Pdx1-Cre*, which were purchased from Shanghai Model Organisms Center, Inc. (www.modelorg.com, Shanghai, China). Primers and PCR conditions for genotyping were described previously (Table S2).^27^ Another two mouse models of acinar-to-ductal metaplasia (ADM) were established by pancreatic ductal ligation (PDL) and caerulein treatment (CAE). All mice were maintained on a mixed background. Male and female pathogen-free athymic nude mice were purchased from the Hunan SJA Laboratory Animal Co., Ltd (Changsha, Hunan, China). All the mice were maintained in specific pathogen-free conditions, and animal experiment procedures were approved by the Ethics Committees or the Institutional Animal Care and Use Committee of the South China University of Technology in compliance with the principles and procedures of the National Institutes of Health Guide for the Care and Use of Laboratory Animals. The immunohistochemical reactivity of FoxM1 was also analyzed, and the quantitation of miRNAs extracted from specific microdissected cells was then determined in triplicate.

### Cell lines and culture condition

The human pancreatic ductal cell lines HPNE and HPDE, and human PDAC cell lines AsPC-1, BxPC-3, CaPan-2, CFPAC-1, HPAC, HPAF-II, MIA PaCa-2, PANC-1, PK-59, PL45, and SW1990, and mouse cell lines including 266-6, MPC-83, and Panc02 were purchased from the American Type Culture Collection (ATCC). All these cell lines were cultivated as adherent monolayers in an appropriate medium, following the Cell Culture Guide from ATCC, and cultured with 10% fetal bovine serum, sodium pyruvate, nonessential amino acids, l-glutamine, and a vitamin solution. The cell lines were subjected to short tandem repeat profiling by ATCC for characterization and authentication. Moreover, the cell lines received from ATCC were used in our laboratory for less than six months after receipt.

### Primary acinar cell separation and culture

Pancreas from wild-type mice was resected and put into Hanks' balanced salt solution with fat tissue removed, and cut into pieces (<1 mm in diameter). Pancreatic tissue fragments were further mixed with soybean trypsin inhibitor (Sigma, CAT# T6522), collagenase P (Roche, CAT# 11213857001), and DNase I (Sigma–Aldrich, CAT# DN25), and shaken to get cell suspension. The primary acini cell suspension was centrifuged and resuspended, mixed with rat tail collagen I (Invitrogen, CAT# A1048301), and cultured in RPMI 1640 supplemented with 1% fetal bovine serum, 1% Pen-Strep, 0.25 mg/mL soybean trypsin inhibitor, and 1 μg/mL dexamethasone at 37 °C and 5% CO_2_. Primary acinar cells were treated with TGF-α (R&D Systems, CAT# 239-A-100) on day 2 for three days, the total RNA was isolated and the levels of miR-23a-3p and -5p were detected.

### Quantitation of miRNAs extracted from cells and pancreatic tissue

Total RNA from PDAC cells, pancreatic cells, and pancreatic tissue were extracted and purified by TRIzol (Invitrogen, CAT# 15596018). All the miRNAs were reverse transcribed into the first strand of cDNA by A-tailing using miRcute Plus miRNA First-Strand cDNA Kit (TIANGEN, CAT# KR211-02), and were further amplified using miRcute Plus miRNA qPCR Kit (SYBR Green) (TIANGEN, CAT# FP411-02) with designed forward primer of miRNAs and universal reverse primer in the kit. The maturation sequences and primer sequences of human and mouse miR-23a-3p and -5p were the same (Table S2). micrON™ mimic, mimic NC, micrOFF™ miRNA inhibitor, and inhibitor NC were custom-designed and purchased from RIBOBIO. micrON™ miR-23a-3p and -5p mimics are chemically synthesized miRNA mimics that mimic high levels of mature miRNA expression in cells. micrOFF™ miRNA inhibitors of miR-23a-3p and -5p were specially modified miRNA inhibitors obtained through chemical synthesis, which inhibit the action of mature miR-23a-3p and -5p by specific binding, and reduce their regulatory effect in cells. Mimic NC and inhibitor NC have no homology with human and mouse genomes and have no influence on the experiment. RNU6B was used as a control, and human and mouse forward primer sequences were the same.

The mRNAs of PDAC cells, pancreatic cells, and pancreatic tissue were reverse transcribed with PrimeScript™ RT Reagent Kit (TaKaRa, CAT# RR047A), and *FOXM1* mRNA was amplified using TB Green® Premix Ex Taq™ (Tli RNaseH Plus) (TaKaRa, CAT# RR420A) with designed forward and reverse primers (Table S2). GAPDH was used as a control.

### Mouse model for PDAC tumorigenesis

An inoculum of 1 × 10^6^ human PDAC cells in 0.1 mL of Hank's balanced salt solution was administered into the right scapular regions of nude mice. The subsequent tumor growth was monitored weekly. Mice with established tumors were euthanized by cervical dislocation either upon reaching a moribund state or on day 35 after injection. The tumors were then collected and weighed.

### Western blot analysis

Whole-cell lysates were subjected to standard western blot analyses using primary antibodies against FOXM1, pancreatic amylase, and CK19 (Beyotime, CAT# P0013B). Peroxidase-linked, species-specific anti-rat, anti-rabbit, or anti-mouse IgG F(ab')2 fragment antibodies were used (Proteintech, CAT# SA00001-2, SA00001-1, SA00001-15). To ensure equal protein sample loading, the same membrane filter was hybridized with either anti-β-actin or GAPDH antibodies. Protein detection was accomplished using the Immobilon™ Western HRP Substrate (Millipore, CAT# WBKLS0500). ImageJ software was utilized to quantify the western blot results, and protein expression was normalized to β-actin or GAPDH. Further details regarding the antibodies can be found in Table S1.

### Plasmids and siRNAs and transient transfection

The plasmids pcDNA3.1-FOXM1 and control vector pcDNA3.1 were procured from Genscript. Lipofectamine 3000 CD transfection reagent (Invitrogen) was employed to transfect the plasmids into PDAC cells. SiRNAs targeting FOXM1 were obtained from Thermo Fisher (Mouse, CAT# 4390771; Human, CAT# 4392420), while positive control GAPDH siRNA (CAT# 4404025) and negative control siRNA (CAT# AM4641) were also utilized. For transient transfection, cells were exposed to varying doses of plasmids, as indicated, for 24–48 h before conducting functional assays.

### Immunofluorescence staining

Primary acinar cells or pancreatic acinar cells were cultured on chamber slides overnight before immunofluorescence staining. Cells were immobilized using 4% paraformaldehyde, followed by permeabilization in 0.1% Triton X-100 in phosphate buffer solution and subsequent blocking with 3% bovine serum albumin for 30 min. Following overnight incubation with primary antibodies against CK19, amylase, and FOXM1, the cells underwent additional incubation with proper secondary antibodies and subsequent staining. Formalin-fixed, paraffin-embedded PDAC specimens or mouse tissues of ADM and pancreatic intraepithelial neoplasia were performed with primary FOXM1, amylase, CK19, α-SMA, and desmin, appropriate secondary antibodies, and DAPI for immunofluorescence staining.

### Luciferase reporter assay

The predicted miR-23a binding site in the 3′-UTR of FOXM1 was cloned into a firefly luciferase reporter plasmid according to the previous report.^26^ The firefly luciferase reporter and a renilla luciferase vector (internal control) were transfected into CFPAC-1 and MIA PaCa-2 human pancreatic cancer cells, and MPC-83 mouse pancreatic cells with miR-23a mimics or NC. The luciferase activity was detected 24 h after transfection using a dual-luciferase reporter assay.^26^

### Statistical analysis

Mean values were compared using Student's *t*-test for paired data. ANOVA was employed for analyzing continuous variables in the two groups' data. Categorical data were analyzed using either Fisher's exact test or the Chi-Square method. Each experiment was independently conducted at least three times, and the results were presented as mean ± standard deviation unless otherwise specified. Analyses were performed using IBM SPSS 21.0 statistical analysis software. All significance was defined at the *p* < 0.05 level (^∗^*P* < 0.05, ^∗∗^*P* < 0.01, ^∗∗∗^*P* < 0.001).

## Results

### The expression levels of miR-23a inversely correlated with FOXM1 in human and mouse PDAC tissues

**Figure 1:**
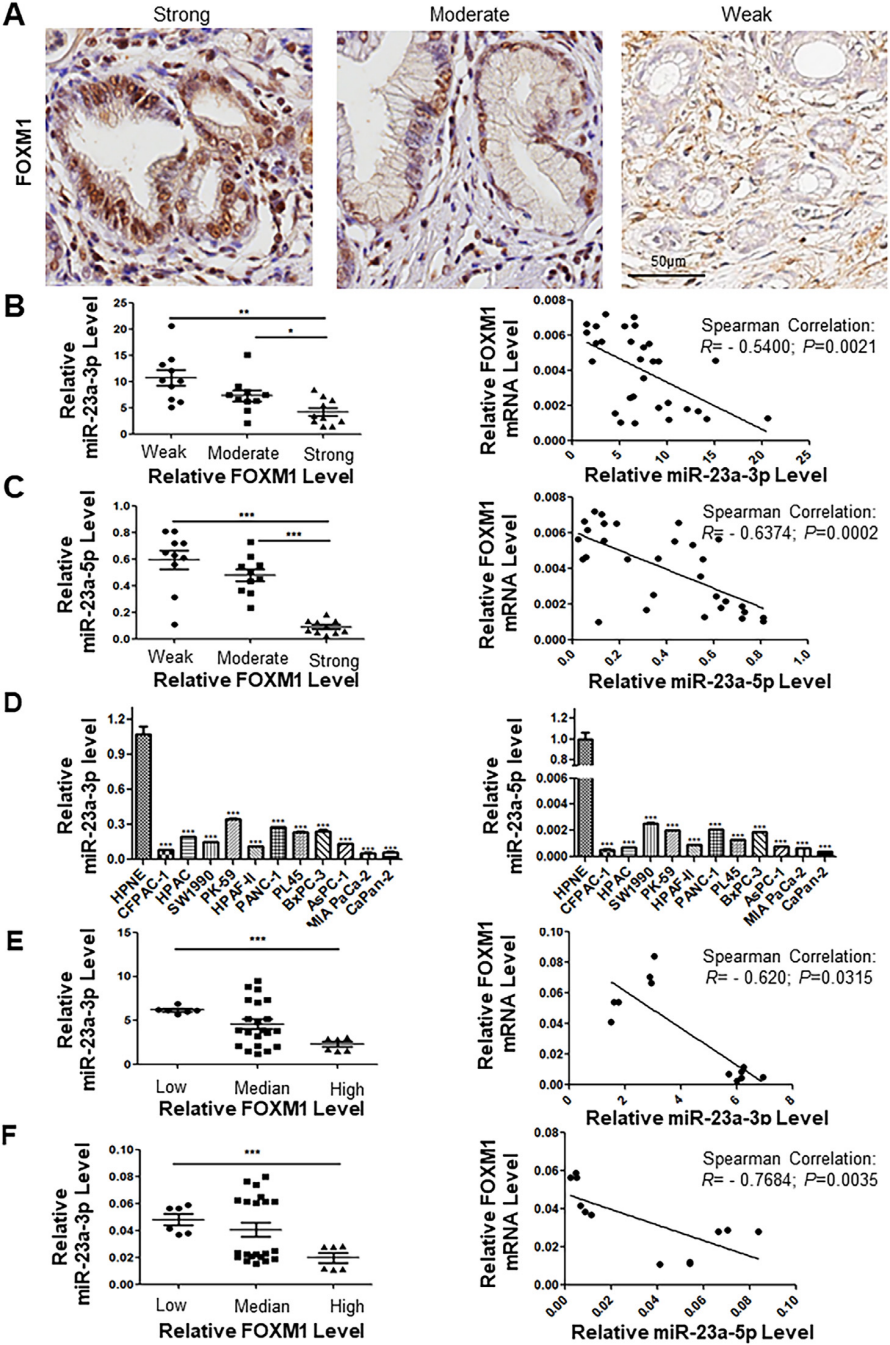
Correlation between miR-23a and FOXM1 expression in human pancreatic ductal adenocarcinoma (PDAC) tissues. **(A)** Total mRNA including miRNA was extracted from distinct cohorts of pancreatic tumor samples with varied levels of FOXM1 expression (negative/weak, moderate, and strong) through laser-assisted microdissection (*n* = 10). **(B, C)** The miR-23a expression levels were quantified using quantitative PCR in triplicate. Notably, an inverse correlation was observed between FOXM1 protein expression and miR-23a-3p (*n* = 30; *r* = −0.5400, *P* = 0.0021) and -5p (*n* = 30; *r* = −0.6374, *P* = 0002) expression. **(D)** Decreased level of both miR-23a-3p and -5p was apparent in human pancreatic cancer cell lines. **(E, F)** The levels of miR-23a-3p and -5p in high FOXM1 mRNA expressing human pancreatic cells were lower than those in low FOXM1 mRNA expressing cells. An inverse correlation was also observed between FOXM1 mRNA expression and miR-23a-3p expression.

Prior investigations have established the up-regulation of FOXM1 in human PDAC cells, and the underlying mechanisms necessitate further exploration.^9^^,^^19^ In our initial investigation, we observed that miR-23a acted as a potential regulator of FOXM1 expression (data not shown). Subsequently, we quantified the expression levels of miR-23a in pancreatic tumor samples exhibiting negative/weak, moderate, and strong FOXM1 expression through laser-assisted microdissection-coupled quantitative PCR (Fig. 1A). The expression levels of miR-23a-3p and -5p in human pancreatic tumor specimens with strong FOXM1 expression (*n* = 10) were significantly lower than those with negative/weak (*n* = 10) and moderate FOXM1 expression (*n* = 10). In addition, our findings revealed significant inverse associations between FOXM1 protein expression and miR-23a-3p (*n* = 30; *r* = −0.5400, *P* = 0.0021) and -5p (*n* = 30; *r* = −0.6374, *P* = 0002) expression (Fig. 1B, C). Consistent results were observed from mouse models of PDAC (Fig. S1A–C). The levels of miR-23a-3p and -5p in pancreatic tumor specimens of KC mice with strong FOXM1 expression (*n* = 10) were lower than those with negative/weak (*n* = 10) and moderate (*n* = 10) FOXM1 expression, and the levels of miR-23a-5p in pancreatic tumor specimens with moderate FOXM1 expression were also lower than those with negative/weak FOXM1 expression. The expression levels of FOXM1 mRNA and protein were also negatively correlated with those of miR-23a-3p (*n* = 30; *r* = −0.5408, *P* = 0.0020) and -5p (*n* = 30; *r* = −0.6939, *P* = 0001).

Moreover, miR-23a expression was significantly decreased in both human and mouse pancreatic cancer cell lines (Fig. 1D; Fig. S1D). Human pancreatic cell lines were divided into three groups according to the expression levels of FOXM1 mRNA. The levels of miR-23a-3p and -5p in high FOXM1 mRNA expressing human pancreatic cell lines (CaPan-2 and HPAF-II) were lower than those in low FOXM1 mRNA expressing cell lines (BxPC-3 and PANC-1). The expression level of FOXM1 mRNA was inversely correlated with the level of miR-23a-3p (*r* = −0.620, *P* = 0.0315) and -5p (*r* = −0.7684, *P* = 0.0035) (Fig. 1E, F). Collectively, our preclinical and clinical data support miR-23a as a negative regulator for the expression of FOXM1 in PDAC.

### miR-23a suppressed pancreatic cancer cell growth and migration *in vitro* and tumorigenesis in animal models

**Figure 2:**
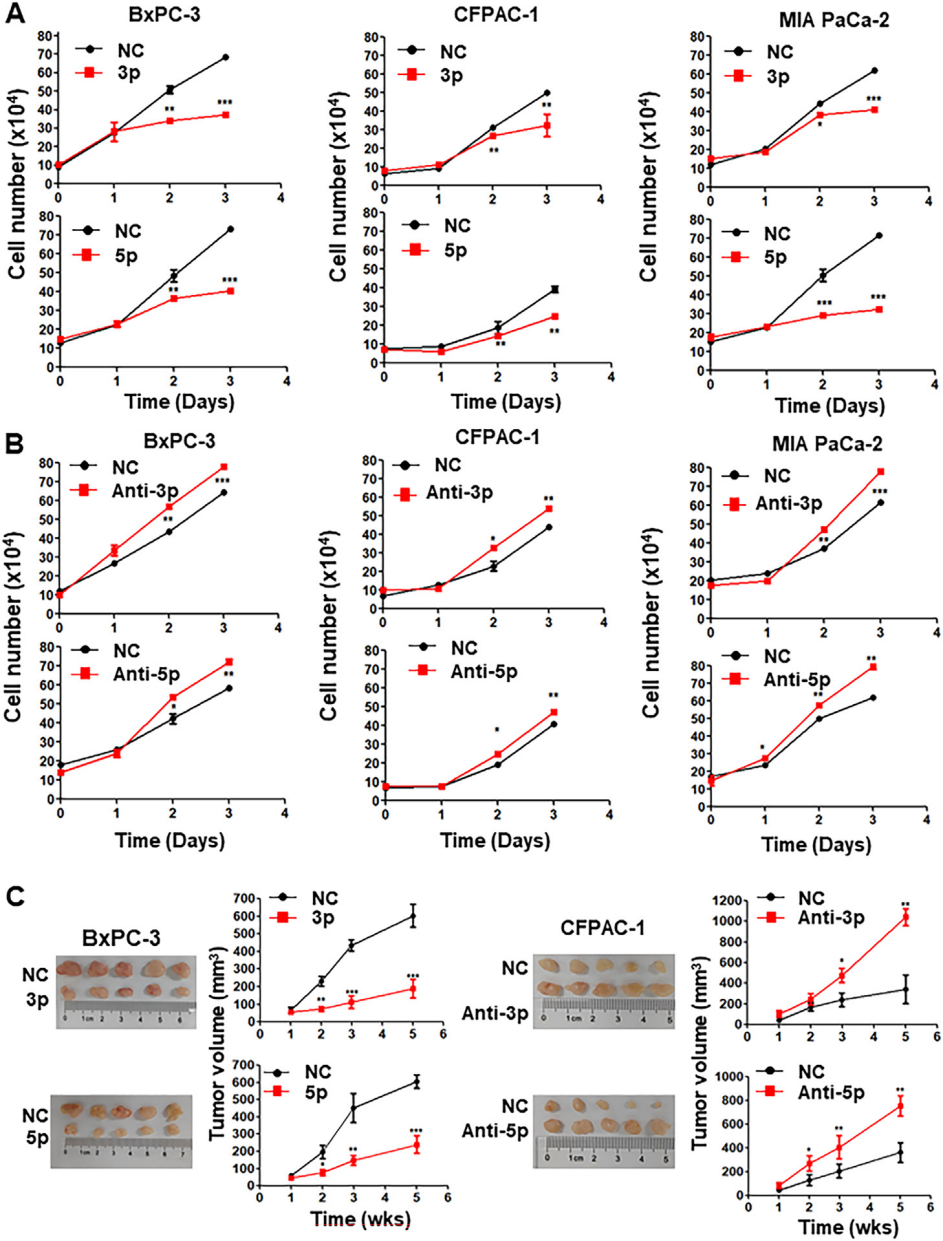
Suppression of pancreatic cancer cell growth by miR-23a *in vitro* and *in vivo*. BxPC-3, CFPAC-1, and MIA PaCa-2 cells were treated with miR-23a-3p, miR-23a-5p, miR-23a-3p inhibitor, or miR-23a-5p inhibitor for indicated times. Cell numbers were determined by CCK8 assay. Note that miR-23a-3p or miR-23a-5p suppressed **(A)** and the inhibitors of miR-23a-3p or miR-23a-5p promoted the growth of human pancreatic cancer cells in BxPC-3, CFPAC-1, and MIA PaCa-2. **(B)** miR-23a suppressed but their inhibitors promoted pancreatic cancer cell tumorigenesis in animal models. **(C)** BxPC-3 and CFPAC-1 human pancreatic cancer cells were transfected with miR-23a-3p, miR-23a-5p, or their inhibitors. The cells were transplanted in the nude mice (*n* = 5) and tumor sizes were measured once every week. Note that overexpression of miR-23a-3p or miR-23a-5p consistently suppressed tumorigenesis when transplanted with BxPC-3, whereas transfection of miR-23a-3p or miR-23a-5p inhibitors promoted tumorigenesis when transplanted with CFPAC-1.

In order to explore the influence of miR-23a expression level on PDAC, we manipulated the expression of miR-23a-3p and -5p in BxPC-3, CFPAC-1, and MIA PaCa-2 cells by transfection of miR-23a mimics or miR-23a inhibitor. It was found that the reintroduction of miR-23a-3p and -5p distinctly hindered the monolayer growth of tumor cells (Fig. 2A), and the cell numbers of BxPC-3, CFPAC-1 and MIA PaCa-2 decreased after transfection with miR-23a-3p. Whereas transfection of miR-23a inhibitors did the opposite (Fig. 2B), the cell numbers of BxPC-3, CFPAC-1, and MIA PaCa-2 increased after transfection with the inhibitor of miR-23a-3p.

Additionally, miR-23a-3p and -5p inhibited the migration of pancreatic cancer cells, while the inhibitors of miR-23a did the opposite (Fig. S2A, B). The migration of BxPC-3 and MIA PaCa-2 cells was inhibited after 48-h treatment by miR-23a-3p and -5p, and was promoted by inhibitors of miR-23a-3p and -5p. However, the migration of CFPAC-1 was inhibited significantly by miR-23-3p and -5p after 24-h treatment, and was significantly promoted by the inhibitors of miR-23-3p and -5p (Fig. S2A, B). To determine whether altered expression of miR-23a impacts the tumorigenesis of human pancreatic cancer cells, BxPC-3 and CFPAC-1 cells were transfected with miR-23a-3p, miR-23a-5p, or miR-23a inhibitor. The cells were transplanted in the nude mice (*n* = 5/group). The overexpression of miR-23a-3p or miR-23a-5p in BxPC-3 resulted in the suppression of tumor cell growth in the subcutis (Fig. 2C), whereas transfection of the inhibitor of miR-23a-3p or miR-23a-5p in CFPAC-1 increased the growth of tumor cells in the subcutis (Fig. 2C). Collectively, the above clinical and experimental evidence substantiates miR-23a as an inhibitor of PDAC growth.

### Decreased expression of miR-23a and increased expression of FoxM1 in mouse premalignant lesions

**Figure 3:**
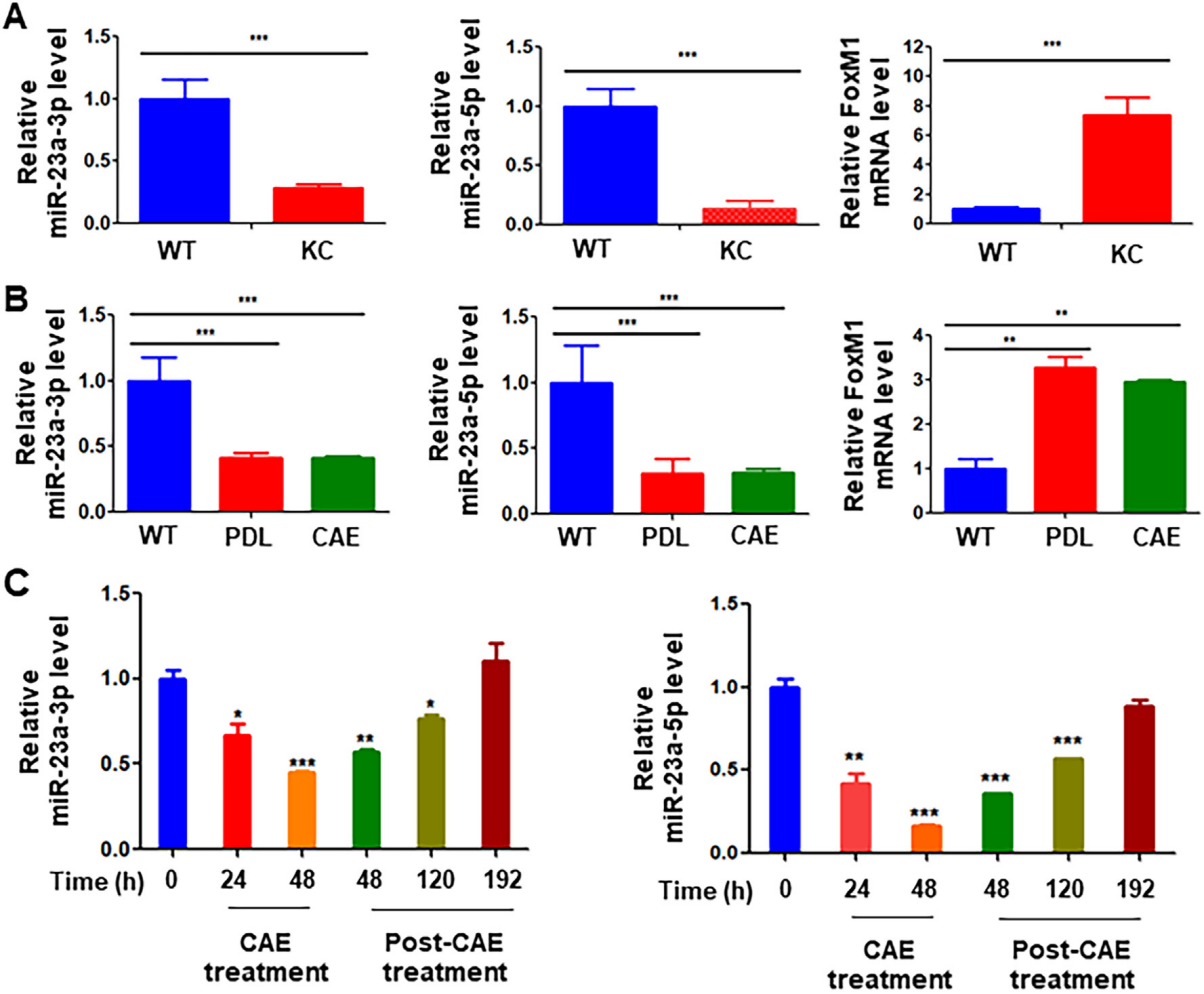
Decreased miR-23a expression in three different mouse models of ADM *in vivo*. **(A, B)***In vivo* ADM induction using three mouse models generated as described in the *Materials and Methods* section, including PDL, CAE, and KC mouse models of pancreatic cancer. FoxM1 and miR-23a expression were determined with quantitative PCR in triplicate. The induction of ADM was accompanied by decreased expression of both miR-23a-3p and miR-23a-5p in KC, PDL, and CAE mouse models (*n* = 5). Note that the increased expression of FoxM1 correlated with a significantly decreased expression of miR-23a-3p and miR-23a-5p. **(C)** Mice were treated with caerulein for two days and miR-23a expression was determined with quantitative PCR 2, 5, and 8 days after the treatment. Caerulein treatment decreased the expression of both miR-23a-3p and miR-23a-5p, while the decreased level of miR-23a-3p and -5p recovered after caerulein treatment was stopped for eight days. PDL, pancreatic ductal ligation; CAE, caerulein treatment; KC, *Pdx1-Cre; LSL-Kras*^*G12D/+*^. ADM, acinar-to-ductal metaplasia.

To further explore the expression of miR-23a in the process of pancreatic carcinogenesis, we performed *in vivo* ADM induction experiments using three mouse models. The induction of ADM was accompanied by decreased levels of both miR-23a-3p and miR-23a-5p in pancreatic tissues from KC, PDL, and CAE mouse models compared with wild-type mice (*n* = 5/group) (Fig. 3A, B). Dramatically, increased expression of FoxM1 correlated with a significantly decreased level of miR-23a-3p and miR-23a-5p in pancreatic tissues from KC, PDL, and CAE mouse models (*n* = 5/group) (Fig. 3A, B). Furthermore, decreased levels of miR-23a-3p and -5p recovered after treatment of caerulein was stopped for eight days (Fig. 3C). The protein expression levels of FOXM1 in most PDAC cell lines were increased, especially in CaPan-2, HPAC, BxPC-3, MIA PaCa-2, PK-59, PL45, and SW1990, compared with pancreatic ductal epithelial cell line HPNE (Fig. S3). These data clearly pointed out that increased expression of FOXM1 is associated with decreased levels of miR-23a, promoting the formation of ADM.

### Induction of ADM decreased the expression of miR-23a expression in acinar cells *in vitro*

**Figure 4:**
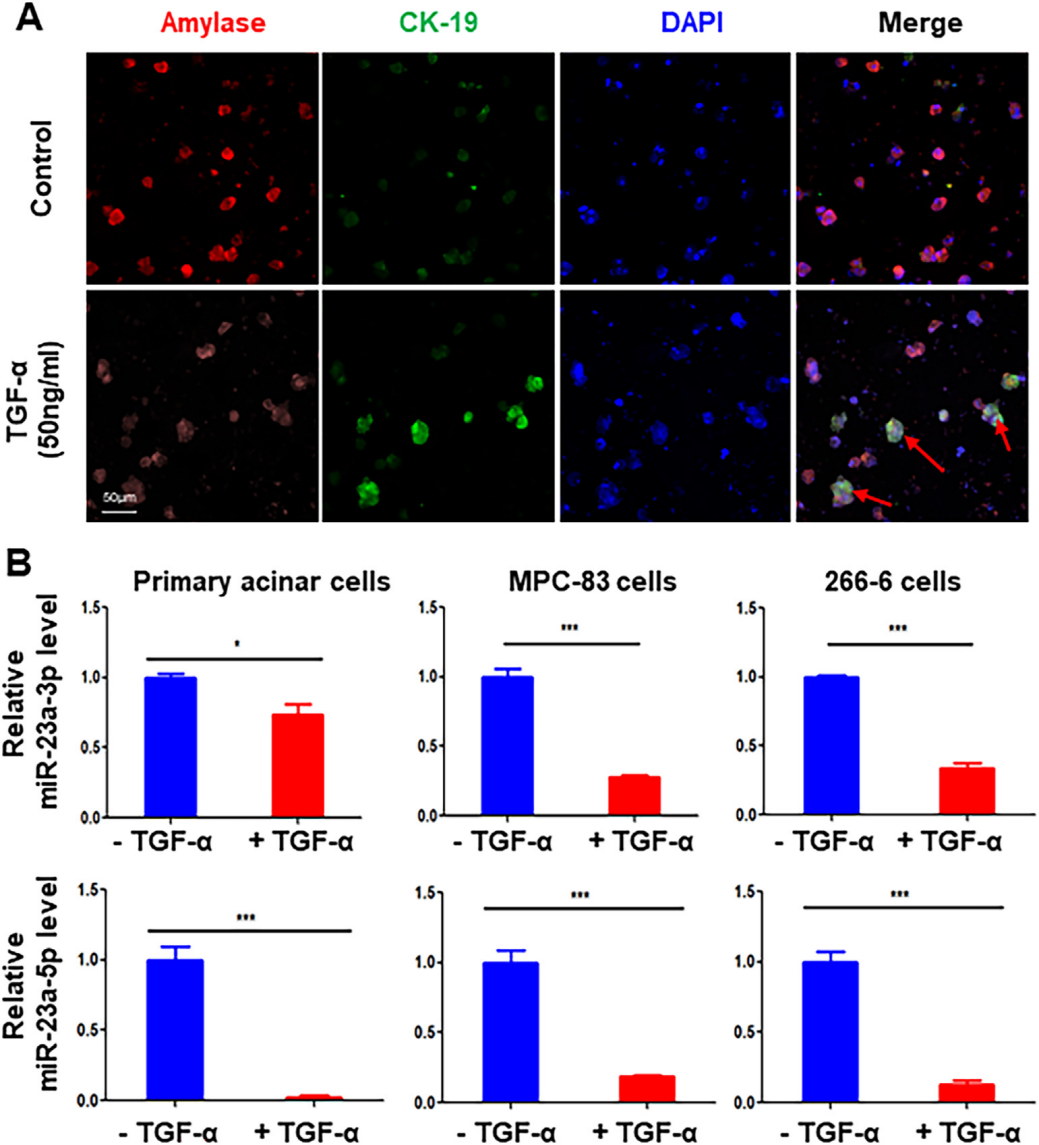
Induction of ADM decreased the expression of miR-23a in primary acinar cells and acinar cells *in vitro*. **(A)** Primary acinar cells were treated with TGF-α for three days, immunofluorescence staining was performed using specific antibodies against CK19 and amylase. Note that the induction of ADM was accompanied by an increased level of CK19 and decreased expression of amylase. **(B)** Primary acinar cells, MPC-83, and 266-6 cells were treated with or without 50 ng/mL TGF-α for three days. miR-23a-3p and miR-23a-5p levels were determined with quantitative PCR in triplicate. Note that induction of ADM correlated with a significantly decreased expression of miR-23a-3p and miR-23a-5p in primary acinar cells and MPC-83 and 266-6 pancreatic cells. ADM, acinar-to-ductal metaplasia.

To investigate the mechanism underlying the increased expression of FOXM1, we performed *in vitro* ADM induction experiments using TGF-α treatment. We performed immunofluorescence staining using specific antibodies against CK19 and amylase. The induction of ADM was accompanied by increased expression of CK19 protein and mRNA and decreased expression of amylase protein and mRNA (Fig. 4A; Fig. S4A, B) in primary acinar cells. Interestingly, increased expression of CK19 correlated with a significantly decreased expression of miR-23a-3p and -5p in primary acinar cells (Fig. 4A, B). Similarly, using MPC-83 and 266-6 pancreatic cells, both miR-23a-3p and -5p decreased after TGF-α treatment, accompanied by increased expression of CK19 protein and mRNA and decreased expression of amylase protein and mRNA. (Fig. S4C, D).

**Figure 5:**
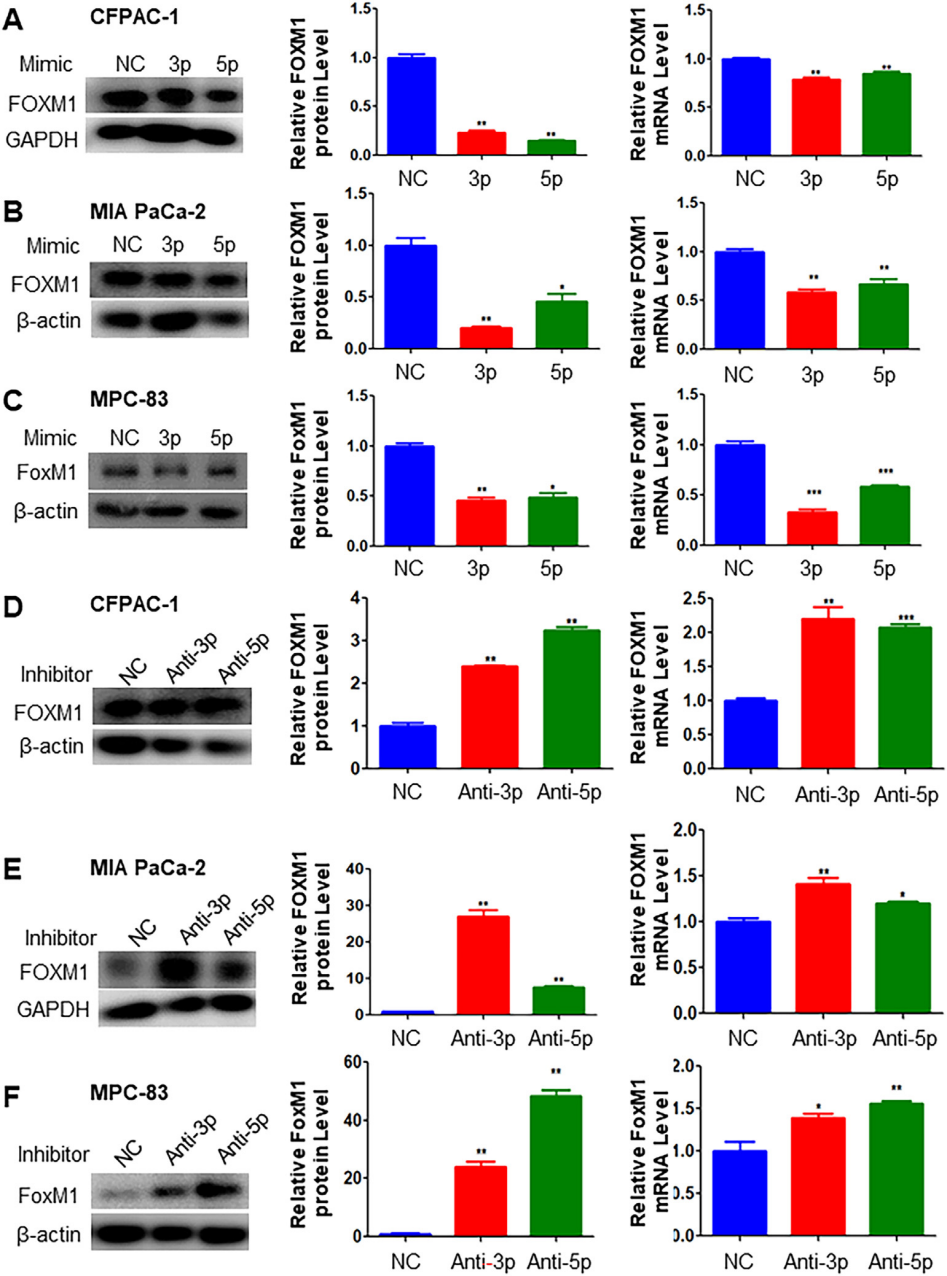
miR-23a down-regulated the expression of FOXM1 in both mouse pancreatic cells and human pancreatic cancer cells. **(A**–**C)** CFPAC-1 and MIA PaCa-2 human PDAC cells and MPC-83 mouse pancreatic cells were treated with either miR-23a-3p or miR-23a-5p for 48 h, and FOXM1 protein and mRNA expression were determined with western blot analyses and quantitative PCR in triplicate. **(D**–**F)** CFPAC-1 and MIA PaCa-2 human PDAC cells and MPC-83 mouse pancreatic cells were treated with inhibitors of either miR-23a-3p or miR-23a-5p for 48 h and FOXM1 protein and mRNA expression were determined with western blot analyses and quantitative PCR. Note that miR-23a-3p and miR-23a-5p inhibited the expression of FOXM1 protein and mRNA in CFPAC-1 and MIA PaCa-2 human pancreatic cancer cells and MPC-83 mouse pancreatic cells, while the inhibitors of miR-23a-3p and miR-23a-5p increased the expression of FOXM1 protein and mRNA in CFPAC-1 and MIA PaCa-2 human pancreatic cancer cells and MPC-83 mouse pancreatic cell.

To further validate the direct regulatory role of miR-23a in FOXM1 expression, we conducted transfections of both mouse and human pancreatic cells with miR-23a-3p or -5p, as well as their respective inhibitors. Clearly, transfection of miR-23a-3p and -5p decreased the expression level of FOXM1 protein and mRNA in CFPAC-1 and MIA PaCa-2 human pancreatic cells and MPC-83 mouse pancreatic cells (Fig. 5A–C), while transfection of miR-23a-3p and -5p inhibitors did the opposite (Fig. 5D–F). Furthermore, elevated FOXM1 expression through the transfection of the FOXM1 expression vector resulted in the down-regulation of miR-23a-3p and -5p expression in 266-6 and MPC-83 mouse pancreatic cells and CFPAC-1 and MIA PaCa-2 human pancreatic cells. Conversely, reduced FOXM1 expression through the transfection of FOXM1 siRNA led to the increase of miR-23a-3p and -5p expression in 266-6 and MPC-83 mouse pancreatic cells and CFPAC-1 and MIA PaCa-2 human pancreatic cells (Fig. S5A, B). The overexpression and knockdown of FOXM1 at mRNA level were confirmed in 266-6, MPC-83, CFPAC-1, and MIA PaCa-2, respectively (Fig. S5C). Therefore, these data clearly show that increased expression of FOXM1 correlated with decreased levels of miR-23a, promoting the formation of ADM, and FOXM1 and miR-23a appear to be mutual negative regulators.

We analyzed the predicted target region of FOXM1 gene of miR-23a and found a predicted site in FOXM1 that could be recognized by miR-23a-5p (Fig. S6A). To determine whether miR-23a could bind to the predicted site of FOXM1 3′-UTR, a fragment of FOXM1 3′-UTR containing the predicted site was cloned into a luciferase reporter plasmid. It was found that the luciferase activity was lower in CFPAC-1, MIA PaCa-2, and MPC-83 when wild-type FOXM1-luc reporter was transfected with miR-23a-5p mimic, than that transfected with wild-type FOXM1-luc reporter or NC (Fig. S6B). The results suggested that miR-23a-5p could bind to 3′-UTR of FOXM1 in the predicted binding site and inhibit FOXM1 expression.

### Increased FOXM1 expression was prevalent in ADM and promoted its formation

**Figure 6:**
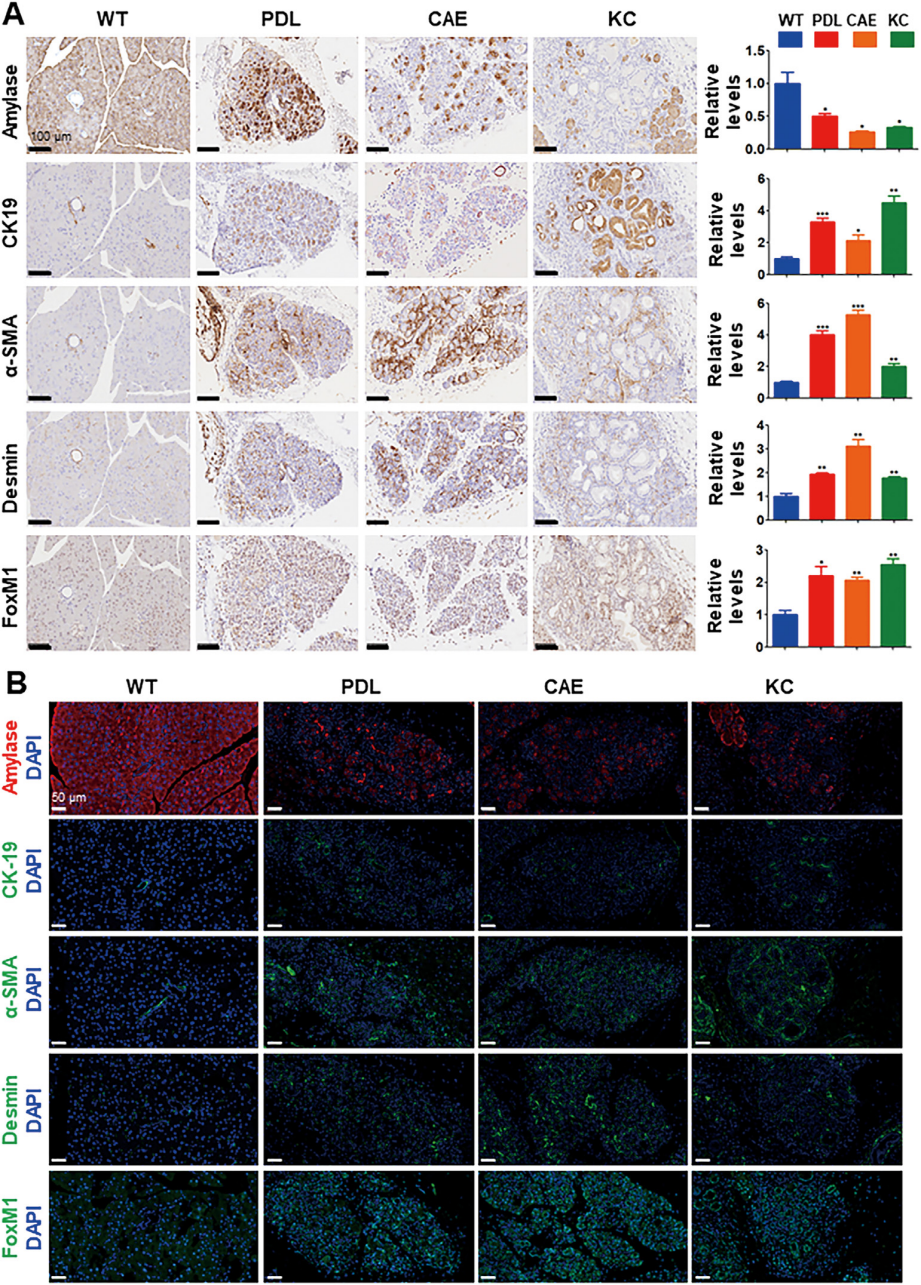
Increased FOXM1 expression in ADM correlated with increased fibrosis in the pancreas. Three mouse models of ADM were generated as described in the *Materials and Methods* section, including PDL, CAE, and KC mouse models. **(A)** Mouse pancreatic tissue sections were prepared and subjected to immunohistochemical staining utilizing specific antibodies against FoxM1, CK19, amylase, α-SMA, and desmin. Relative expressions of those proteins were determined. The expression level of CK19, α-SMA, desmin, and FoxM1 increased, and the expression level of amylase decreased in PDL, CAE, and KC mouse models compared with wild-type (WT) mouse model (*n* = 5). **(B)** Mouse pancreatic tissue samples were sectioned and subjected to immunofluorescence staining with specific anti-FoxM1, anti-CK19, anti-amylase, anti-α-SMA, and anti-desmin antibodies. PDL, pancreatic ductal ligation; CAE, caerulein treatment; KC, *Pdx1-Cre;LSL-Kras*^*G12D/+*^; ADM, acinar-to-ductal metaplasia.

We determined whether increased FOXM1 expression occurred in ADM, the major premalignant lesion of PDAC. Three mouse models of ADM were used, including PDL, CAE, and KC mouse models of PDAC. We then performed both immunohistochemistry and immunofluorescence staining experiments using specific antibodies against FoxM1, CK19, amylase, α-SMA, and desmin (Fig. 6A, B). The induction of ADM was accompanied by increased expression of CK19 and FoxM1 and decreased expression of amylase in PDL, CAE, and KC mouse models compared with wild-type mice (*n* = 5). Interestingly, increased expression of FoxM1 correlated with a significantly increased expression of α-SMA and desmin in PDL, CAE, and KC mice (*n* = 5), suggesting that increased expression of FOXM1 promoted the formation of extracellular matrix, such as fibrosis formation, thus promoting PDAC development and progression.

Finally, to explore the potential clinical implications of miR-23a and FOXM1 as biomarkers or therapeutic targets in PDAC patients. We analyzed the impacts of the expression levels of miR-23a and FOXM1 on overall survival in PDAC patients using Gene Expression Omnibus and TCGA databases. The overall survival of PDAC patients decreased when the expression level of FOXM1 was higher (Fig. S7A). However, there was no significant difference in survival of patients with low or high expression levels of miR-23a (Fig. S7B).

## Discussion

The present study provided comprehensive evidence substantiating the crucial involvement of miR-23a in the abnormal activation of FOXM1 in PDAC tissue samples and PDAC cell lines. Firstly, we found an inverse correlation of the expression level between miR-23a and FOXM1 in PDAC. Secondly, miR-23a expression was significantly diminished in PDAC cells, and restoring miR-23a expression led to the down-regulation of FOXM1 in these cells. Thirdly, increased FOXM1 expression suppressed miR-23a expression. Fourthly, miR-23a inhibited PDAC cell growth and migration. Collectively, these novel discoveries unveil a significant upstream mechanism playing an important role in the abnormal activation of miR-23a/FOXM1 signaling in PDAC cells, and underscore its crucial role in influencing PDAC pathogenesis.

miR-23a, as a member of the miR-23a-27a-24-2 cluster, is located at chromosome 19 of the human genome and plays an important role in the regulation of gene expression.^28^^,^^29^ Various studies have examined the expression change and clinical influence of this miRNA, which plays significant roles in development, fibrotic diseases, autoimmune diseases, inflammation, and cancer.^29^, ^30^, ^31^, ^32^, ^33^ However, the expression and functions of miR-23a in oncogenesis in various tissues remain highly controversial. miR-23a demonstrates inhibitory effects in melanoma, nephroblastoma, osteosarcoma, prostate carcinoma, and leukemia,^34^, ^35^, ^36^, ^37^ while it exhibits promotive effects in lung cancer, liver cancer, and colorectal cancer.^38^, ^39^, ^40^ In some types of cancer, miR-23a appears to exert both promotive and inhibitory effects.^41^^,^^42^ These observations may be attributed to the distinct biological characteristics of different tumors and the intricate regulatory mechanisms involving miRNA. In this study, we conducted a comprehensive assessment of the miR-23a expression difference in pancreatic tumor cells and their corresponding normal cells from PDAC tissue samples obtained by laser-assisted microdissection. The miR-23a expression of both PDAC and normal ductal cells was quantified. The statistical analyses revealed a significant down-regulation of miR-23a expression in PDAC cells, which strongly correlated with the malignant characteristics of PDAC. We also demonstrated that reintroducing miR-23a through genetic engineering suppressed PDAC cell growth in both *in vitro* and *in vivo* experimental models, using various cell lines and manipulating gene expression levels. These clinical and experimental results robustly support miR-23a as a PDAC growth suppressor.

However, the observable function of miR-23a in various tumor types appears to be contingent on the specific context, implying that its role is subject to regulation by distinct effectors and/or signaling pathways.^28^^,^^43^^,^^44^ FOXM1 stands as a pivotal transcription factor with significance in tissue morphogenesis and cellular propagation. Contemporary investigations have showcased an up-regulated expression of FOXM1 across an array of malignancies, encompassing pancreatic tumors.^10^^,^^12^^,^^45^, ^46^, ^47^ Our extensive research endeavors have firmly established FOXM1 as a crucial central gene that efficiently relays upstream regulations to downstream effectors (such as cyclin D1, vascular endothelial growth factor, and caveolin-1), which promotes angiogenesis, development, invasion, and metastasis in pancreatic cancer.^9^^,^^19^, ^20^, ^21^^,^^26^^,^^48^^,^^49^ Broadly, FOXM1 expression is overexpressed in tumors, regenerating tissue, and developing/progenitor tissue, whereas the overall expression of miRNAs is commonly diminished in these scenarios.^50^, ^51^, ^52^ In the present study, we have observed a cause-and-effect relationship between diminished miR-23a expression and heightened FOXM1 expression in PDAC cells. Specifically, we manipulated miR-23a expression genetically, which directly influenced FOXM1 expression. Additionally, we assessed the functional consequences of altered miR-23a expression and found its dependence on FOXM1 expression. Furthermore, we identified miR-23a as a pivotal miRNA associated with FOXM1 overexpression in PDAC cells. Notably, we consistently observed reciprocal miR-23a and FOXM1 expression changes in PDAC cells, providing substantial evidence to underscore the clinical relevance and importance of miR-23a′s negative regulation of FOXM1 expression in these cells. This observation bears particular significance, as the miR-23a expression is generally down-regulated in PDAC cells. Consequently, the modification of miR-23a/FOXM1 signaling represents a novel mechanism responsible for the aberrant activation of FOXM1 in PDAC cells, further emphasizing the relevance of FOXM1 expression in PDAC development and progression. Moreover, it introduces miR-23a as a potential novel target for PDAC treatment.

To further analyze the mechanisms underlying the dysregulation of miR-23a in pancreatic cancer development, we quantitated miR-23a expression in normal pancreatic epithelial, ADM, and pancreatic intraepithelial neoplasia in mouse models. Our findings incontrovertibly revealed a significant reduction in miR-23a expression within ADM and pancreatic intraepithelial neoplasia cells compared with normal cells. We also found an increased FOXM1 expression occurred in ADM, the major premalignant lesion of PDAC. We used three mouse models of ADM including PDL, CAE, and KC mouse models of PDAC. The induction of ADM was accompanied by increased expression of CK19 and FOXM1 and decreased expression of amylase. Interestingly, increased expression of FOXM1 linked to a significantly increased expression of α-SMA and desmin, suggesting that increased expression of FOXM1 promoted the formation of extracellular matrix, such as fibrosis formation, which is consistent with the role of FOXM1 in pulmonary fibrosis and renal fibrosis.^53^^,^^54^ Furthermore, pancreatic fibrosis promotes the development and progression of PDAC.^55^ Therefore, our findings suggest that down-regulation of miR-23a expression is an early event during pancreatic cancer initiation and progression.

In conclusion, the miR-23a/FOXM1 signaling axis is important in PDAC initiation and progression. In this study, miR-23a was identified as a critical tumor-suppressive microRNA in PDAC cells, consistently noting its frequent down-regulation in these cells. Functionally, miR-23a exerted inhibitory effects on PDAC cell growth and migration by negatively modulating FOXM1 expression, thereby attenuating the FOXM1 signaling pathway. Further exploration of the mechanisms underlying the dysregulation of miRNA expression in pancreatic cancer cells promises to provide valuable insights, advancing our comprehension of PDAC progression. Moreover, this investigation holds potential for the development of novel biomarkers facilitating PDAC diagnosis and prognosis for patients with early or late stages of PDAC, and will also help identify potential therapeutic targets for PDAC treatment.

## Author contributions

LL performed experiments and analyzed and interpreted the data. TC, XL, JA, SY, YZ, and FG conducted experiments. FW and JH performed experiments and analyzed the data. KX provided supervision and funding and contributed to manuscript writing. TJ also provided supervision and funding and made significant contributions to manuscript writing, including reviewing and editing the manuscript before its submission and actively participating in content discussions. All authors read and approved the final manuscript.

## Funding

This work was supported by grants from Guangzhou Ruiqian Biotech Company (China) (No. 20230330 to TJ), the 10.13039/501100001809National Natural Science Foundation of China (No. 82072632), Guangzhou Municipality Bureau of Science and Technology, Guangzhou, China (No. 202102010033), and the 10.13039/501100003453Natural Science Foundation of Guangdong Province, China (No. 2022A1515012585 to KX).

## Conflict of interests

The authors declare no conflict of interests.
