# Supplementary material for: Down-regulation of microRNA-23a promotes pancreatic ductal adenocarcinoma initiation and progression by up-regulation of FOXM1 expression
Source: Genes Dis. 2023 Dec 22;11(5):101203. doi: 10.1016/j.gendis.2023.101203 (PMC11252794; doi:10.1016/j.gendis.2023.101203)
Supplement: Multimedia component 1 [file mmc1.pdf]

Supplementary Materials

1. Supplementary Tables

Table S1. Detailed information of antibodies

Antibody name	Company	Cat.	Application
FoxM1 (D3F2B) Rabbit mAb	CST	20459	WB
FOXM1 Polyclonal antibody	Proteintech	13147-1-AP	WB
FOXM1 antibody (G-5)	Santa	sc-376471	IHC
FOXM1	Sigma	ABE1000	IF
CK19	DSHB	TROMA-III	WB, IF
CK19	Abcam	ab52625	IHC
Amylase	Invitrogen	PA5-117115	WB
Amylase	Santa	sc-46657	IHC, IF
α-SMA	Abcam	ab124964	IHC, IF
Desmin	Abcam	ab227651	IHC, IF

Table S2. PCR primer sequences for analyses of gene expression

Gene name	Species	Forward primer (5´ to 3´)	Reverse primer (5´ to 3´)
FOXM1	Human	tgcagctagggatgtgaatcttc	ggagcccagtccatcagaact
FoxM1	Mouse	gccatgatacagtttgccatc	agagaaaggttgtgacgaatagag
GAPDH	Human	caggaggcattgctgatgat	gaaggctggggctcattt
GAPDH	Mouse	ggttgtctcctgcgacttca	tggtccagggtttcttactcc
LSL-Kras (Wild type)	Mouse	tgtctttccccagcacagt	ctgcatagtacgctataccctgt
LSL-Kras (Mutant-G12D)	Mouse	gcaggtcgagggacctaata	ctgcatagtacgctataccctgt
Pdx1-Cre	Mouse	cctggactacatcttgagttgc	aggcaaattttggtgtacgg
mir-23a-5p	Human, Mouse	tcaggggttcctggggatgggattt	/
mir-23a-3p	Human, Mouse	tgcgcatcacattgccagggatttcc	/
RNU6B	Human, Mouse	gcaaggatgacacgcaaattc	/

Note, both GAPDH and RNU6B were used as internal controls.

2. Supplementary Figures

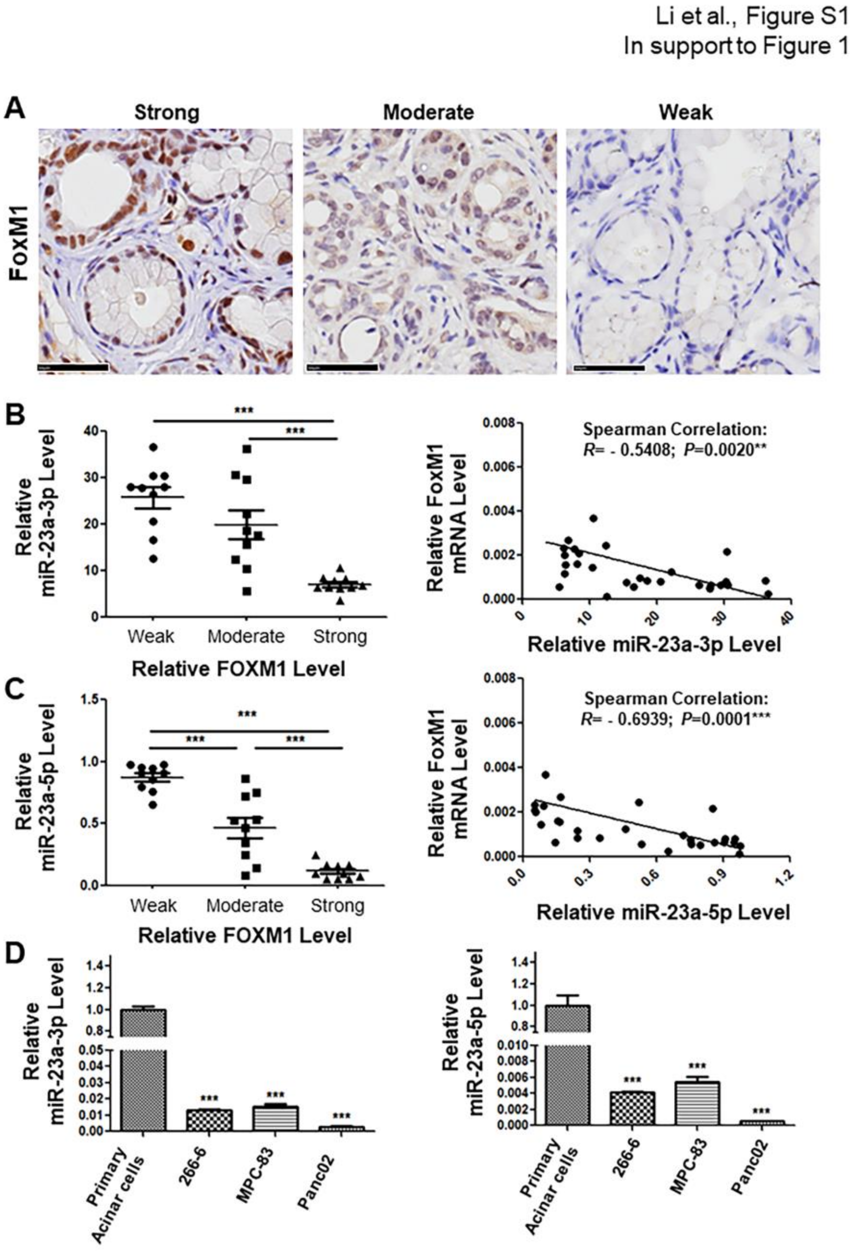

Figure S1. Correlation between the expression of miR-23a and FoxM1 in mouse pancreas tumor tissues. (**A**), Total mRNA including miRNA was collected from groups of pancreatic tumor specimens with negative/weak (n=10), moderate (n=10), and strong (n=10) nuclear FoxM1 expression by using LMD. (**B** & **C**), The levels of miR-23a expression were measured by qPCR. Note the inverse relationship between the expression of nuclear FoxM1 mRNA and protein and that of miR-23a-3p (n=30) and -5p (n=30). (**D**), Decreased expression of both miR-23a-3p and -5p was apparent in 266-6 and MPC-83 mouse pancreatic cells and Panc02 mouse pancreatic cancer cells as compared with that in primary acinar cells.

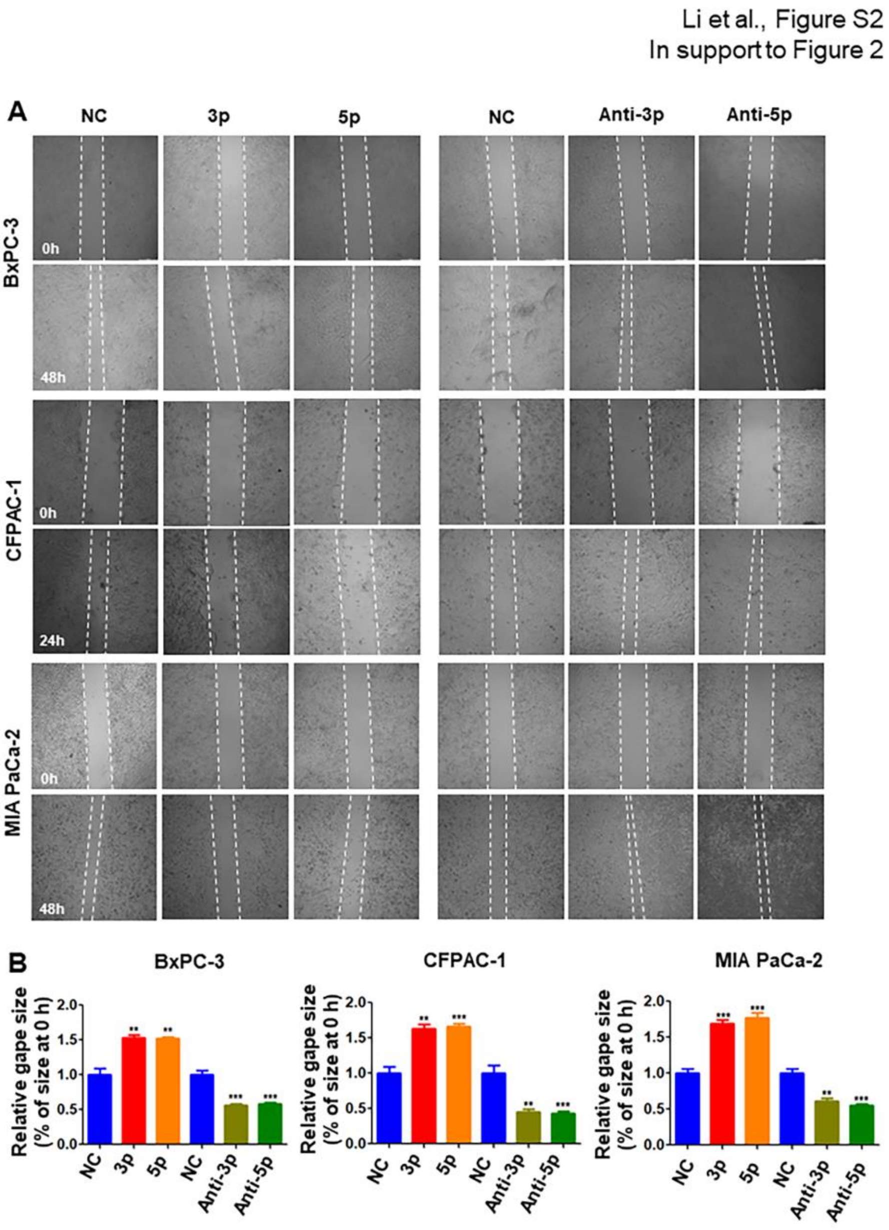

Figure S2. Suppression of pancreatic cancer cell migration by miR-23a *in vitro*. BxPC-3, CFPAC-1 and MIA PaCa-2 cells were treated with miR-23a-3p, miR-23a-5p, miR-23a-3p inhibitor or miR-23a-5p inhibitor for indicated times. Cell migration ability was determined by wound healing assay (**A**). Note that miR-23a-3p or miR-23a-5p suppressed and the inhibitors of miR-23a-3p or miR-23a-5p promoted the migration of human pancreatic cancer cells BxPC-3, CFPAC-1 and MIA PaCa-2 (**B**).

Li et al., Figure S3
In support to Figure 3

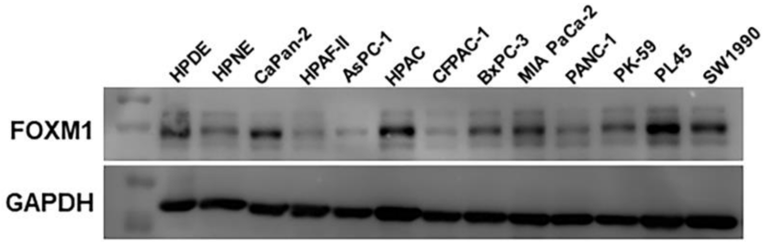

Figure S3. Increased expression level of FOXM1 in PDAC cell line *in vitro*. The protein levels of FOXM1 in most of PDAC cell lines (CaPan-2, HPAF-II, AsPC-1, HPAC, CFPAC-1, BxPC-3, MIA PaCa-2, PANC-1, PK-59, PL45, and SW1990) were increased, especially in CaPan-2, HPAC, BxPC-3, MIAPaCa-2, PK-59, PL45, and SW1990, compared with pancreatic ductal epithelial cell line HPNE.

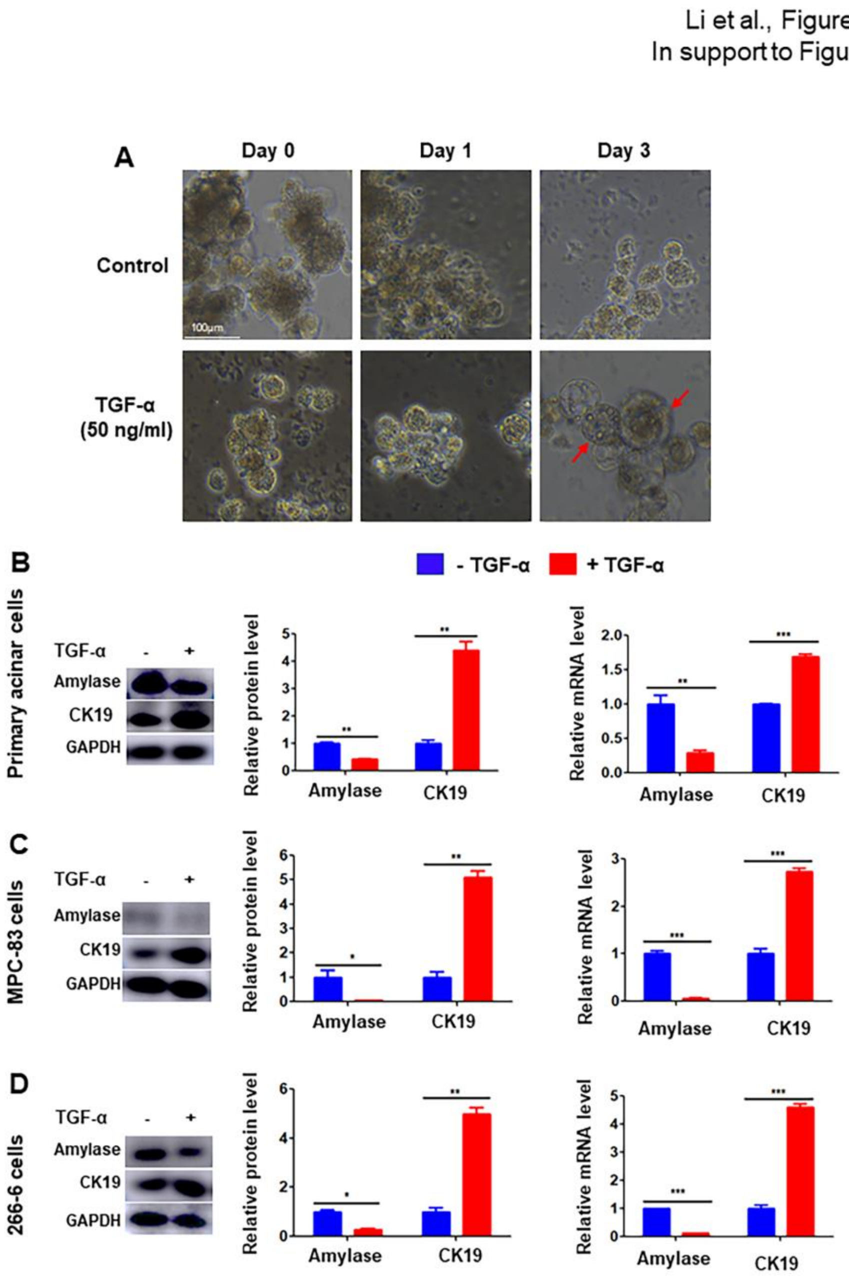

Figure S4. Induction of ADM decreased the expression of miR-23a expression in acinar cells *in vitro*. *In vitro* ADM induction experiment: primary mouse acinar cells were isolated from mice and cultured *in vitro* containing 50 ng/ml TGF-α. (**A**), morphological observation. Note that duct-like structure was formed 3 days after TGF-α treatment. Primary acinar cells (**B**), MPC-83 pancreatic cells (**C**) and 266-6 pancreatic cells (**D**) were treated with TGF-α for 3 days, and gene expressions were determined using Western blot analyses and qPCR. Note that the induction of ADM accompanied by increased expression of CK19 protein and mRNA, but decreased expression of amylase protein and mRNA in primary acinar cells, MPC-83 and 266-6 pancreatic cells.

Li et al., Figure S5
In support to Figure 5

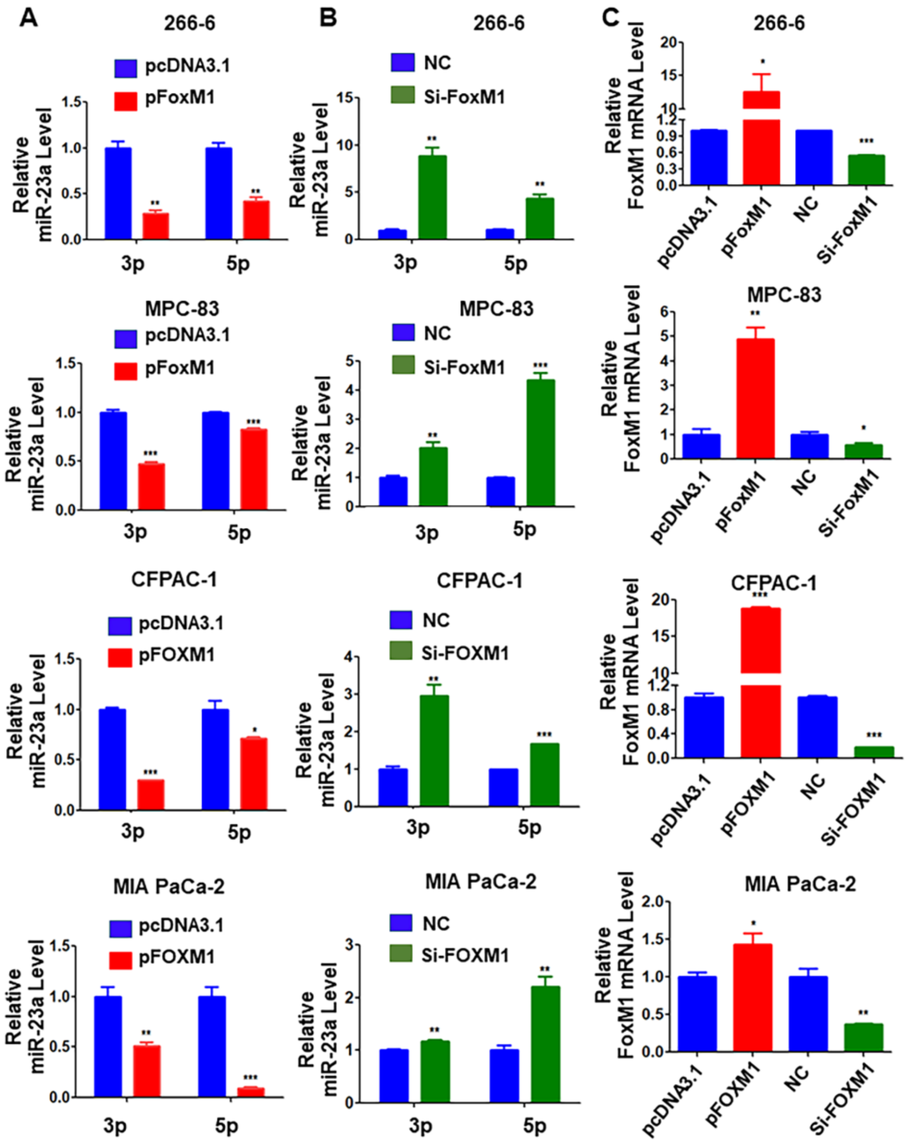

Figure S5. FOXM1 downregulated the expression of miR-23a in both mouse pancreatic cells and human PDAC cells. 266-6 and MPC-83 mouse pancreatic cells and CFPAC-1 and MIA PaCa-2 human PDAC cells were transfected with either FOXM1 expression vector “pFOXM1” or its control “pcDNA3.1” (**A**) or FOXM1 siRNA “si-FOXM1” or its control “NC” (**B**) for 48 hrs. Both miR-23a-3p and miR-23a-5p were determined using qPCR. Note that increased expression of FOXM1 inhibited the expression of miR-23a-3p and -5p in both 266-6 and MPC-83 mouse pancreatic cells and CFPAC-1 and MIA PaCa-2 human pancreatic cells, while knockdown of FOXM1 increased the expression of both miR-23a-3p and -5p in 266-6, MPC-83, CFPAC-1 and MIA PaCa-2. (**C**), The overexpression and knockdown of FOXM1 at mRNA levels were confirmed in 266-6, MPC-83, CFPAC-1 and MIA PaCa-2.

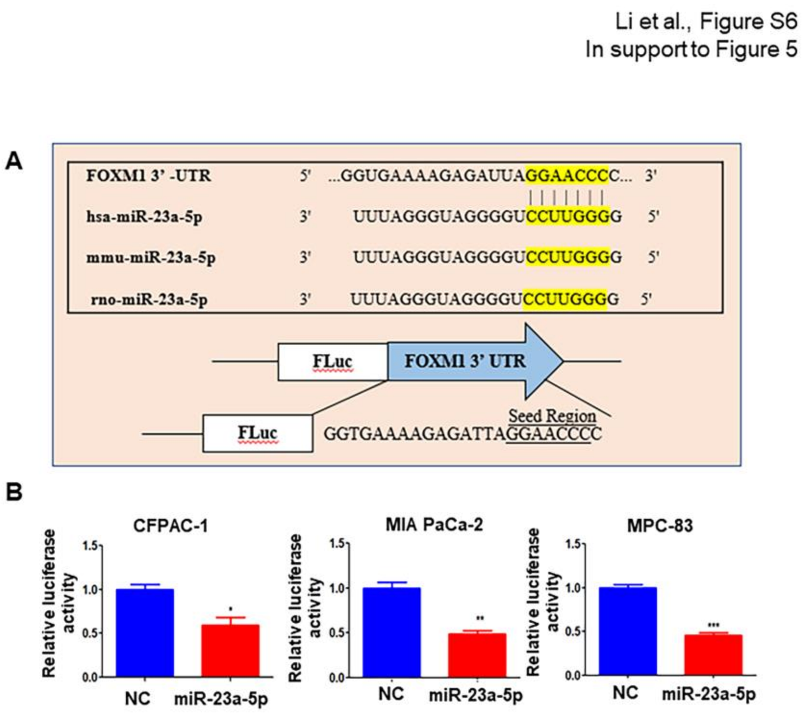

Figure S6. MiR-23a directly targets FOXM1 mRNA. (**A**), Predicted target region of human FOXM1 3'-UTR by miR-23a-5p was analyzed and showed; miR-23a-5p-binding regions were conserved among human, mouse and rat, and the seed region of the predicted target region was also showed. (**B**), Luciferase activity was determined after transfection with FOXM1 dual-luciferase reporter vector with miR-23a mimics or NC into CFPAC-1, MIA PaCa-2 and MPC-83 cells. The luciferase activity was lower in CFPAC-1, MIA PaCa-2 and MPC-83 when co-transfected wild-type FOXM1-luc reporter with miR-23a-5p, than that transfected wild-type FOXM1-luc reporter with NC.

Li et al., Figure S7
In support to Figure 6

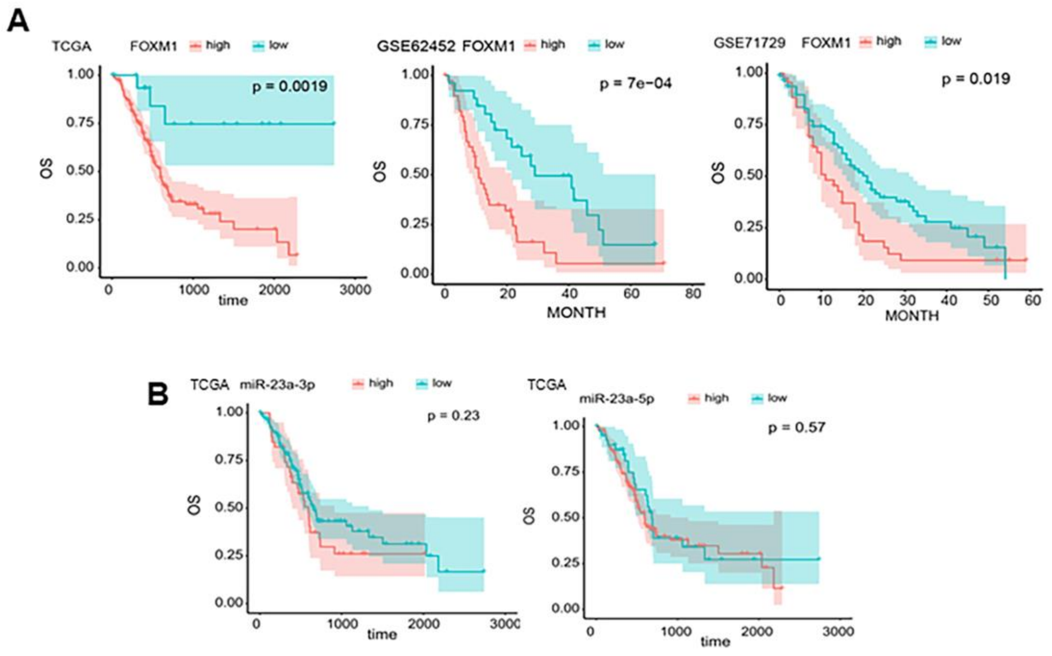

Figure S7. Increased FOXM1 expression associated with poor patient survival in PDAC patients. (**A**), Overall survival (OS) curves of PDAC patients with low and high expression of FOXM1 from different databases, and the overall survival of PDAC patients decreased when the expression level of FOXM1 was higher. (**B**), OS curves of PDAC patients with low and high expression of miR-23a from TCGA database showed that, there was no significant difference in survival of patients with low or high expression levels of miR-23a.
